# Ensemble Modeling for Aromatic Production in *Escherichia coli*

**DOI:** 10.1371/journal.pone.0006903

**Published:** 2009-09-04

**Authors:** Matthew L. Rizk, James C. Liao

**Affiliations:** Department of Chemical and Biomolecular Engineering, University of California Los Angeles, Los Angeles, California, United States of America; German Cancer Research Center, Germany

## Abstract

Ensemble Modeling (EM) is a recently developed method for metabolic modeling, particularly for utilizing the effect of enzyme tuning data on the production of a specific compound to refine the model. This approach is used here to investigate the production of aromatic products in *Escherichia coli*. Instead of using dynamic metabolite data to fit a model, the EM approach uses phenotypic data (effects of enzyme overexpression or knockouts on the steady state production rate) to screen possible models. These data are routinely generated during strain design. An ensemble of models is constructed that all reach the same steady state and are based on the same mechanistic framework at the elementary reaction level. The behavior of the models spans the kinetics allowable by thermodynamics. Then by using existing data from the literature for the overexpression of genes coding for transketolase (Tkt), transaldolase (Tal), and phosphoenolpyruvate synthase (Pps) to screen the ensemble, we arrive at a set of models that properly describes the known enzyme overexpression phenotypes. This subset of models becomes more predictive as additional data are used to refine the models. The final ensemble of models demonstrates the characteristic of the cell that Tkt is the first rate controlling step, and correctly predicts that only after Tkt is overexpressed does an increase in Pps increase the production rate of aromatics. This work demonstrates that EM is able to capture the result of enzyme overexpression on aromatic producing bacteria by successfully utilizing routinely generated enzyme tuning data to guide model learning.

## Introduction

The manipulation of the enzymatic reactions which make up metabolic networks is at the heart of metabolic engineering. However, due to the complex and highly-interconnected nature of these networks, it is sometimes not a trivial process to understand what the effect of an enzymatic perturbation will be, or vice versa, what enzymatic perturbations are necessary to yield a desired effect. Flux distribution is often controlled by multiple enzymes in the network [1]–[10], indirectly linked to the pathways of interest. Thus, it is desirable to develop mathematical models to describe, understand, and predict network behavior. Through the development of such models, one gains the ability to generate a set of testable hypotheses for system behavior.

In typical kinetic modeling, kinetic parameters are determined in order to best fit the time-dependent metabolite concentration data obtained from experiment, using a wide variety of kinetic rate expressions. However, these types of data are rare and are not commonly generated in a typical strain improvement process. On the other hand, enzyme overexpressions or knockouts are commonly used in strain development, and the effects of enzyme expression tuning on product formation or substrate consumption are the typical readouts. To our knowledge, such data are difficult to incorporate into modeling, particularly when the results are semi-quantitative, since the fold-changes of enzyme overexpression are rarely measured.

Recently, the Ensemble Modeling (EM) approach was developed for the modeling of metabolic networks [11]–[13]. The detailed EM framework has been previously published [11], but few biological examples were used as validation. EM has been used to study a real biological example for the production of lysine [13], and EM has been compared to other modeling methodologies in more detail [12]. In this approach, rather than focusing on the development of a kinetic model that fits the dynamic metabolite concentration data, we seek to utilize data that captures the effect of enzyme tuning on the steady state production flux to guide model development. This type of data is not typically utilized in modeling endeavors, and does not result in a change in the stoichiometry of the network. However, the effect that an enzyme's overexpression has on the system's steady state flux can unveil some knowledge regarding how control over the flux is distributed throughout the metabolic network, and can thus be used to aid in model development. EM is related to the insightful application of Metabolic Control Analysis (MCA) to metabolic systems with uncertain kinetic parameters in that a random sampling of kinetics was used for analysis [14], [15]. However, EM does not require the MCA relationships which are derived based on a linearized system. Other uses of sampling in metabolic analyses was reviewed previously [16].

**Figure 1:**
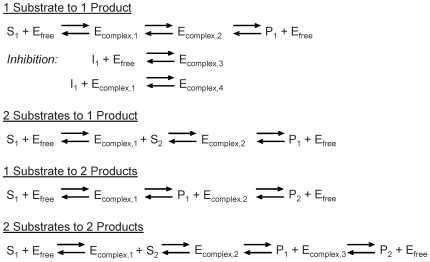
The elementary reaction mechanisms. Mechanisms used for the variety of different metabolic reactions. Reactions modeled as 1 substrate to 1 product: HPr, EIIA, Pgi, Tpi, Gpm, Eno, Pgl, Rpe, Rpi, Pta, Ppc, Fum, Sdh, recycle reactions of ATP, NADH and NADPH. 2 substrate to 1 product reactions: EIIBC, AroG^fbr^. 1 substrate to 2 products: EI, Fba, Pfl. 2 substrates to 2 products: Pfk, Gap, Pgk, Zwf, Gnd, Tkt, Tal, Pyk, Pps, Ack, Mdh.

**Figure 2:**
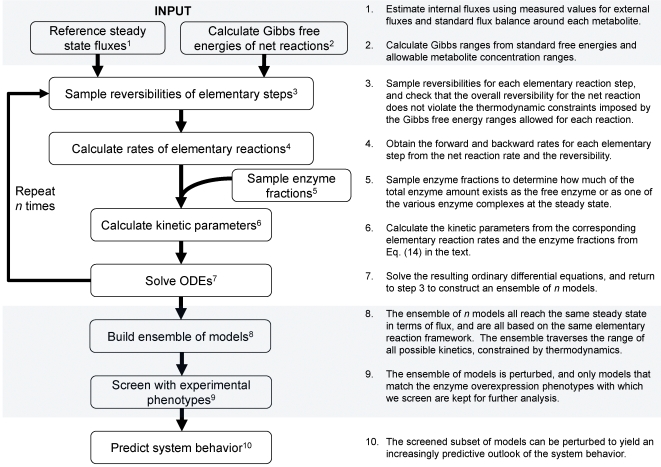
Algorithm for Ensemble Modeling. The algorithm is illustrated on the left, while the steps are described in detail on the right.

In EM, initially, an ensemble of models that all reach the reference steady state in terms of flux distribution is constructed. These models span the space of kinetics allowable by thermodynamic constraints, and are based on elementary reactions, which are the most fundamental and general kinetic descriptions for enzymatic reactions [6], [17]. EM describes the kinetics using a set of elementary reactions, as shown in **Figure 1**, which can be transformed into a set of log-linear equations. This transformation requires mass-action kinetics for the reactions, which is not valid if enzyme concentrations are not explicitly considered. However, through the use of elementary reactions at the enzyme level, the log-linear transformation can be completed, while still preserving the intrinsic non-linear behavior in enzyme kinetics, thus preserving the true biological mechanism and benefiting from the mathematical tractability. This framework does not rely on a local linearization of the system, and one is free to perform and determine the effect of large perturbations on the network. In lieu of dynamic metabolite data, which can be difficult to obtain, the EM approach uses phenotypic data to screen possible models. Phenotypic data can include the effects of enzyme overexpressions or knockouts on the production rate of any products or byproducts. Such data are routinely generated in strain design efforts. Through this approach, one does not attempt to acquire detailed kinetic parameters that fit the time-dependent metabolite data, but rather capture phenotypes that are dependent on changes in the enzyme levels in the network. Also important is the ability of EM to be driven by the goal of, and ability to, learn from experimental results regarding the phenotypes obtained by enzyme perturbations. Rather than assessing the control properties of the system, or fitting dynamic metabolite concentrations, the focus of EM is to rely on existing experimental data to drive strain design. Through this knowledge feedback loop, we can further constrain our subset of models and thus refine our predictions of system behavior. The algorithm for EM is illustrated in **Figure 2**.

In this work, we implement the EM approach to the production of aromatic amino acids in *Escherichia coli*. Aromatic compounds are of substantial industrial importance, with many uses and high rates of production, including the aromatic amino acids and other derived compounds such as indigo, quinic acid and catechol [18], [19]. L-Tryptophan is primarily used as a feed and food additive, and has other pharmaceutical applications [20]. The estimated production rate of L-Tryptophan is 500 ton/year [21]. L-Phenylalanine is primarily used for the production of the artificial sweetener aspartame [20], [22], and as a nutraceutical, a flavor enhancer, and an intermediate for pharmaceutical production [20], with an estimated production of 8,000 ton/year [21]. L-Tyrosine is used in the production of the anti-Parkinson's drug L-DOPA, and as a dietary supplement [20], and is produced at a smaller scale of about 120 ton/year [21].

In *E. coli* and many other organisms, the production of aromatic compounds begins with the condensation reaction between phosphoenolpyruvate (PEP) and erythrose-4-phosphate (E4P) to form 3-deoxy-D-arabinoheptulosonate-7-phosphate (DAHP). This reaction, catalyzed by DAHP synthase (AroG), is the committed step and the most tightly regulated reaction in the common aromatic amino acid pathway [20]. DAHP has often been used as an index for the capability of aromatics production [19], [22]–[27].

We choose the production of aromatic amino acids in *Escherichia coli* as a system to demonstrate the applicability of EM because this system is an example of both kinetic and stoichiometric limitations that takes place in metabolic systems. Stoichiometric analysis shows that the theoretical yield of DAHP production from glucose in the wildtype *E. coli* is 43% (mol/mol) [19], [22]. This relatively low theoretical yield is due to the fact that *E. coli* cannot recycle pyruvate back to PEP during glycolytic growth. Thus, when PEP is converted to pyruvate during glucose transport via the phosphotransferase system (PTS), the pyruvate generated cannot be used in the aromatic synthesis, causing a decrease in yield. In order to increase the yield, either pyruvate has to be recycled back to PEP via PEP synthase (Pps) [22], or use a PEP-independent glucose transport system [19], [25]. Once pyruvate is recycled or a PEP-independent transport system is used, the theoretical yield increased to 86% (mol/mol). However, when Pps is overepressed in *E. coli*, there is no change in DAHP yield. On the other hand, overexpression of Tkt effectively increases the yield to close to 43%. Overexpression of both Pps and Tkt then increases the yield to 86%, approaching the theoretical limit [19], [22]. This example indicates that Tkt is the first limiting enzyme. Once this bottleneck is removed, then Pps can demonstrate its effect. This type of kinetic behavior cannot be captured using either stoichiometric models or a direct flux comparison between flux states.

Here we use EM to study the production of DAHP from glucose in *E.coli*, utilizing existing data from the literature to screen the ensemble of models. By using data that has been reported for the overexpression of transketolase (Tkt), transaldolase (Tal) and phosphoenolpyruvate synthase (Pps), we can screen the original ensemble of models to a subset that accurately describes the kinetic phenomena that is at work in this system. The final screened ensemble of models demonstrates the property that the E4P metabolite pool is initially limiting, and only after this limitation is lifted, does the PEP pool become important. Thus, we can correctly predict that only after Tkt is overexpressed does the overexpression of Pps play a role in an increased rate of DAHP production. This work thus demonstrates that EM is able to capture properties of metabolic networks in a real biological system through the utilization of enzyme tuning data to drive model development.

## Results

### The DAHP Metabolic Network

**Figure 3:**
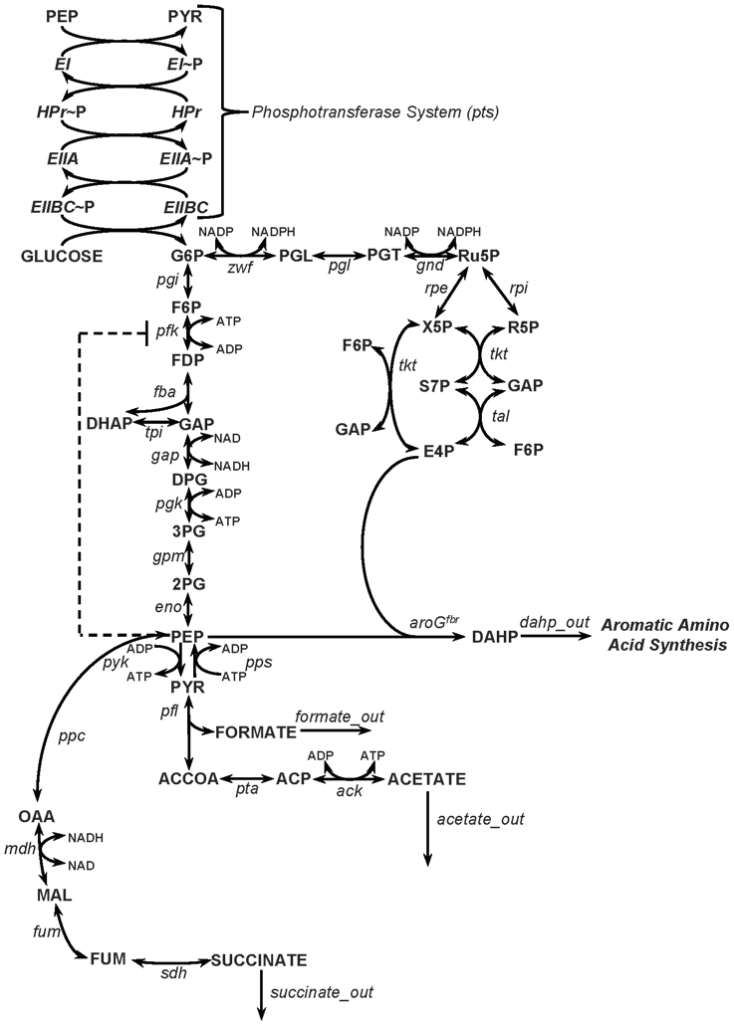
The metabolic network for the production of aromatic precursor 3-deoxy-D-arabino-heptulosonate-7-phosphate (DAHP). Metabolites are denoted by capital letters, while enzyme abbreviations are in italics. The metabolite PEP feedback inhibits the enzyme phosphofructokinase (*pfk*). In the studied system, 2-dehydro-3-deoxyphosphoheptonate aldolase (*aroG*) had already been made feedback resistant, denoted by superscript “fbr”.

The metabolic network for the production of DAHP is depicted in **Figure 3**. This network includes the PTS for glucose uptake, glycolysis pathway, pentose phosphate pathway, Krebs cycle, the formate and acetate production pathways, and the pathway for the synthesis of DAHP. The PTS is modeled as a series of phosphorylation steps [11]. First, enzyme I (EI) uses PEP as the phosphoryl donor, thus converting PEP to pyruvate and phosphorylating histidine protein (HPr). Next, HPr phosphorylates enzyme IIA (EIIA), which in turn phoshorylates enzyme IIBC (EIIBC). Finally, in the last step, EIIBC transfers the phosphate to glucose, thus converting the glucose to glucose-6-phosphate (G6P). Further, phosphofructokinase (Pfk) is feedback inhibited by phosphoenolpyruvate (PEP). This network consists of 37 net reactions and 34 metabolites. The full list of abbreviations used in this network can be found in **Table 1**.

**Table 1:** Abbreviations for metabolites and enzymes.

Metabolite Name	Metabolite Symbol	Enzyme Name	Enzyme Symbol
2-phosphoglycerate	2PG	acetate transport	acetate_out
3-phosphoglycerate	3PG	acetate kinase	ack
acetyl-CoA	ACCOA	2-dehydro-3-deoxyphosphoheptonate aldolase	aroG
acetate	ACETATE	DAHP transport	dahp_out
acetyl phosphate	ACP	enzyme I	EI
adenosine diphosphate	ADP	enzyme IIA	EIIA
adenosine triphosphate	ATP	enzyme IIBC	EIIBC
3-deoxy-D-arabino-heptulosonate-7-phosphate	DAHP	enolase	eno
dihydroxy acetone phosphate	DHAP	fructose biphosphate aldolase	fba
1,3-biphosphoglycerate	DPG	formate transport	formate_out
erythrose-4-phosphate	E4P	fumarase	fum
fructose-6-phosphate	F6P	glyceraldehyde 3-phosphate dehydrogenase	gap
fructose-1,6-biphosphate	FDP	6-phosphogluconate dehydrogenase	gnd
formate	FORMATE	phosphoglycerate mutase	gpm
fumarate	FUM	histidine protein	HPr
glucose-6-phosphate	G6P	malate dehydrogenase	mdh
glyceraldehyde-3-phosphate	GAP	phosphofructokinase	pfk
b-D-glucose	GLUCOSE	pyruvate formate lyase	pfl
malate	MAL	phosphogluco isomerase	pgi
nicotinamide adenine dinucleotide	NAD	phosphoglycerate kinase	pgk
nicotinamide adenine dinucleotide reduced	NADH	6-phosphogluconolactonase	pgl
nicotinamide adenine dinucleotide phosphate	NADP	phosphoenolpyruvate carboxylase	ppc
nicotinamide adenine dinucleotide phosphate reduced	NADPH	phosphoenolpyruvate synthase	pps
oxaloacetate	OAA	phosphate acetyltransferase	pta
phosphate group	P	phosphotransferase system	pts
phosphoenolpyruvate	PEP	pyruvate kinase	pyk
6-phosphogluconolactone	PGL	ATP recycle	recATP
6-phosphogluconate	PGT	NADH recycle	recNADH
pyruvate	PYR	NADPH recycle	recNADPH
ribose-5-phosphate	R5P	ribulose-5-phosphate 3-epimerase	rpe
ribulose-5-phosphate	Ru5P	ribulose-5-phosphate isomerase	rpi
sedoheptulose-7-phosphate	S7P	succinate dehydrogenase	sdh
succinate	SUCCINATE	succinate transport	succinate_out
xylulose-5-phosphate	X5P	transaldolase	tal
		transketolase	tkt
		triose phosphate isomerase	tpi
		glucose-6-phosphate dehydrogenase	zwf

### Obtaining Steady State Fluxes

For the production of DAHP, the external fluxes of the system (glucose uptake and the secretion of DAHP, succinate, acetate and formate) have been reported [19]. However, it can be seen that even when the external fluxes are determined, there is an additional degree of freedom at the metabolite glucose-6-phosphate (G6P), where the flux coming into the system is split between glycolysis (via Pgi) and the pentose phosphate pathway (via Zwf). To account for this additional degree of freedom in determining the steady state flux, we calculate the flux map for a variety of glycolysis:pentose phosphate pathway split ratios (25∶75, 50∶50, 75∶25 and 95∶5) and carry out the EM approach using each of these varying flux maps to examine the effect of the split ratio on the modeling results. In addition, recycle reactions are included to allow the cell to dispose of extra energy (ATP) or reducing power (NADH, NADPH). Each of the steady state fluxes for the various split ratios are reported in **Table 2**.

**Table 2:** Net flux (mmol/g DCW/hr) of reference state for various glycolysis vs pentose pathway split ratios.

Glycolysis∶Pentose Split	25∶75	50∶50	75∶25	95∶5
acetate_out	1.408	1.517	1.625	1.712
ack	1.408	1.517	1.625	1.712
aroG	0.260	0.260	0.260	0.260
dahp_out	0.260	0.260	0.260	0.260
EI	1.300	1.300	1.300	1.300
EIIA	1.300	1.300	1.300	1.300
EIIBC	1.300	1.300	1.300	1.300
eno	1.928	2.037	2.145	2.232
fba	0.888	0.997	1.105	1.192
formate_out	1.408	1.517	1.625	1.712
fum	0.260	0.260	0.260	0.260
gap	1.928	2.037	2.145	2.232
gnd	0.975	0.650	0.325	0.065
gpm	1.928	2.037	2.145	2.232
HPr	1.300	1.300	1.300	1.300
mdh	0.260	0.260	0.260	0.260
pfk	0.888	0.997	1.105	1.192
pfl	1.408	1.517	1.625	1.712
pgi	0.325	0.650	0.975	1.235
pgk	1.928	2.037	2.145	2.232
pgl	0.975	0.650	0.325	0.065
ppc	0.260	0.260	0.260	0.260
pps	0.006	0.011	0.017	0.022
pta	1.408	1.517	1.625	1.712
pyk	0.114	0.228	0.342	0.433
recATP	2.557	2.773	2.990	3.163
recNADH	1.668	1.777	1.885	1.972
recNADPH	1.950	1.300	0.650	0.130
rpe	0.563	0.347	0.130	−0.043
rpi	0.412	0.303	0.195	0.108
sdh	0.260	0.260	0.260	0.260
succinate_out	0.260	0.260	0.260	0.260
tal	0.412	0.303	0.195	0.108
tkt (1)	0.412	0.303	0.195	0.108
tkt (2)	0.152	0.043	−0.065	−0.152
tpi	0.888	0.997	1.105	1.192
zwf	0.975	0.650	0.325	0.065

### Construction of the Initial Ensemble

For each glycolysis∶pentose pathway split ratio, an ensemble of 1500 models was constructed using Matlab (Mathworks, Natick, MA) on an Intel (Santa Clara, CA) Pentium 4 processor running Microsoft (Redmond, WA) Windows XP. The total computational time to develop and perturb each ensemble to obtain the overexpression phenotypes of interest was approximately 18 hours. In addition to the steady state fluxes, the other input into the algorithm is the standard Gibbs free energies for each reaction, which are listed in **Table 3**. The reactions are then broken down into their elementary steps, as described in Methods. The way that each reaction in the network is modeled is described in **Figure 1**. The reversibilities for each elementary step are sampled uniformly from zero to one, and the thermodynamic compliance of the reaction is checked using Eq. (13). If the reversibilities are determined to be outside the constraints imposed by thermodynamics, they are resampled. The enzyme fractions for each set of elementary reactions are uniformly sampled from zero to one, and each of the fractions relating to enzyme *i* are rescaled such that there sum is equal to one.

**Table 3:** Overall reactions and free energies.

Reaction	Overall Equation	Inhibitor	Free Energy (kcal/mol)
*acetate_out*	ACETATE -->out	–	−3.5
*ack*	ACP+ADP --> ACETATE+ATP	–	−4.7
*aroG*	PEP+E4P --> DAHP	–	−17.9
*dahp_out*	DAHP --> out	–	−3.5
*EI*	PEP --> PYR+P	–	−6.45
*EIIA*	P --> P	–	−0.1
*EIIBC*	P+GLUCOSE --> G6P	–	−6.45
*eno*	2PG --> PEP	–	−0.2
*fba*	FDP --> DHAP+GAP	–	1.1
*formate_out*	FORMATE --> out	–	−3.5
*fum*	MAL --> FUM	–	1.3
*gap*	GAP+NAD --> DPG+NADH	–	4.2
*gnd*	PGT+NADP --> Ru5P+NADPH	–	−0.8
*gpm*	3PG --> 2PG	–	−2.2
*HPr*	P --> P	–	−0.1
*mdh*	OAA+NADH --> MAL+NAD	–	−4.8
*pfk*	F6P+ATP --> FDP+ADP	PEP	−4.5
*pfl*	PYR --> FORMATE+ACCOA	–	−2.5
*pgi*	G6P --> F6P	–	−2.5
*pgk*	DPG+ADP --> 3PG+ATP	–	4.7
*pgl*	PGL --> PGT	–	−13.3
*ppc*	PEP --> OAA	–	−11.7
*pps*	PYR+ATP --> PEP+ADP	–	−3.6
*pta*	ACCOA --> ACP	–	−3.9
*pyk*	PEP+ADP --> PYR+ATP	–	−8.4
*recATP*	ATP --> ADP	–	−0.1
*recNADH*	NADH --> NAD	–	−0.1
*recNADPH*	NADPH --> NADP	–	−0.1
*rpe*	Ru5P --> X5P	–	−0.1
*rpi*	Ru5P --> R5P	–	0.7
*sdh*	FUM --> SUCCINATE	–	−0.7
*succinate_out*	SUCCINATE --> out	–	−3.5
*tal*	S7P+GAP --> F6P+E4P	–	−0.6
*tkt (1)*	X5P+R5P --> S7P+GAP	–	0.9
*tkt (2)*	X5P+E4P --> F6P+GAP	–	−0.6
*tpi*	DHAP --> GAP	–	0.2
*zwf*	G6P+NADP --> PGL+NADPH	–	−0.9

### Perturbation and Screening of the Ensemble

In our system of interest for the production of DAHP, an important metabolic kinetic phenomenon is at work. The synthesis of DAHP draws from the pools of two metabolites, PEP and E4P, from different areas of the metabolic network. Thus, how these pools are balanced, a kinetic property, is the key to increased DAHP production. It has been demonstrated that the E4P metabolite pool is the limiting metabolite in DAHP synthesis, and that only after this limitation is lifted do perturbations in enzyme levels that increase the PEP pool size yield an increase in production. It has been reported that the overexpression of Tkt [19], [22]–[26] and Tal [27], which increase the E4P pool, lead to an increase in the DAHP production rate and yield from glucose. While the overexpression of both of these enzymes increased the production rate, it was found that overexpression of Tkt had a stronger effect than the overexpression of Tal [27]. Further, the overexpression of Tkt shows a negligible impact on the glucose uptake rate [19], [22], [26]. The overexpression of Pps alone, which would increase the PEP pool, had no effect on DAHP production [22]. However, when both Tkt and Pps were overexpressed simultaneously, the combined effect of these overexpressions was far greater than the single overexpression of either Tkt or Pps [22], [26]. All of the phenotypes are summarized in **Table 4**.

**Table 4:** Summary of literature used for screening phenotypes.

Target Gene(s)	Phenotype	Reference
transketolase (*tkt*)	*tkt* overexpression increases DAHP production rate	Draths et al. (1992), Flores et al. (1996), Gosset et al. (1996), Baez et al. (2001)
transketolase (*tkt*)	*tkt* overexpression has no change on glucose uptake	Patnaik & Liao (1994), Patnaik et al. (1995), Gosset et al. (1996)
transaldolase (*tal*)	*tal* overexpression increases DAHP production rate	Lu & Liao (1997)
phosphoenolpyruvate synthase (*pps*)	*pps* overexpression has no change on DAHP production rate	Patnaik & Liao (1994)
*tkt* & *tal*	*tkt* overexpression gives a larger increase than tal overexpression	Lu & Liao (1997)
*tkt* & *pps*	*tkt* & *pps* simultaneous overexpression increases DAHP production rate	Patnaik & Liao (1994), Gosset et al. (1996)

After constructing the ensemble of models for each of the split ratios, each model was perturbed by overexpressing Tkt, Tal and Pps two-fold, one at a time. After perturbation of the ensemble, the models that did not match the literature phenotypes were screened out of the ensemble. For the phenotypes where overexpression led to an increase in DAHP production (Tkt and Tal), models that exhibited any increase in the DAHP production rate were kept. For the phenotype of Pps overexpression leading to no change in the DAHP rate, only models that did not exceed an increase or decrease of 10% of the maximum DAHP rate increase for that ensemble were kept. For the glucose uptake rate being unchanged with Tkt overexpression, only models that exhibited less than a 10% change in the glucose uptake rate were retained. To screen for Tkt overexpression increasing the DAHP rate more than Tal overexpression, only models where the DAHP rate when Tkt is overexpressed was greater than the DAHP rate when Tal was overexpressed were retained.

**Figure 4:**
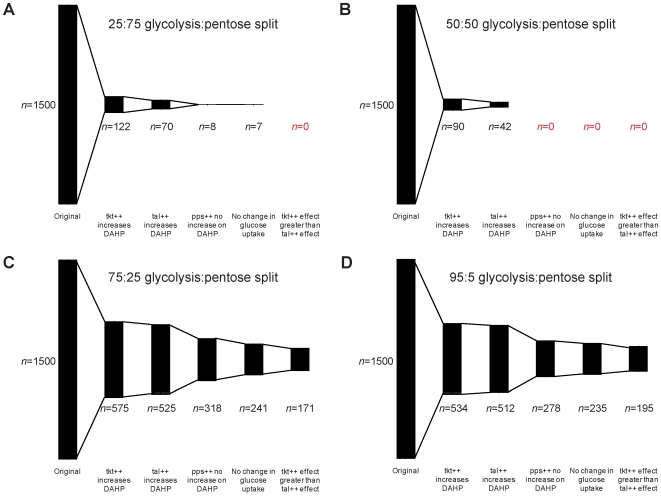
Results of screening for each of the reported literature phenotypes. Screening of phenotypes from Table 4, using each of the four glycolysis∶pentose phosphate pathway split ratios. For the two fluxes with the highest proportion of the flux through the pentose-phosphate pathway (panels A&B), zero models in the ensemble were able to match all of the experimental phenotypes. For the two flux distributions with the highest flux through glycolysis (panels C&D), approximately one-tenth of the original ensemble matched each of the screening phenotypes. The size of each column represents the number of models remaining after the screening step indicated on the x-axis.

The results of the screening are illustrated in **Figure 4**. In screening for the overexpression of Tkt leading to an increase in the DAHP production rate, 122, 90, 575 and 534 models were retained for the 25∶75, 50∶50, 75∶25 and 95∶5 split ratios, respectively. From this reduced subset of models, screening for those in which Tal overexpression also leads to an increase in the DAHP production, led to the retention of 70, 42, 525, and 512 models for the 25∶75, 50∶50, 75∶25 and 95∶5 split ratios, respectively. It should be noted that higher fractions of models are retained in each successive step. This result suggests that the ensemble becomes more and more predictive. Ideally, when the model is truly predictive, 100% of the ensemble will be retained between screening steps and the phenotypic screening will converge to the same ensemble of models. **Figs. 4C & 4D** illustrate this phenomenon. On the other hand, when no model is retained after screening, at least one of the underlying assumptions is invalid. This phenomenon is illustrated in **Figs. 4A & 4B**, where incorrect glycolysis∶pentose pathway split ratios gave no acceptable model.

**Figure 5:**
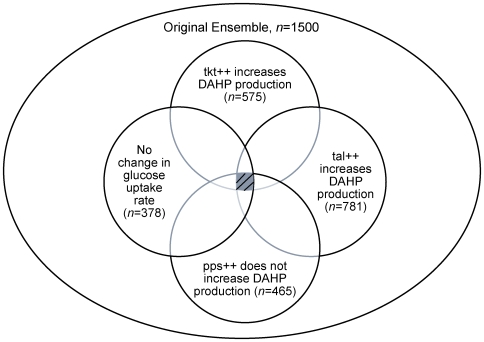
Illustration of screening the ensemble of models using different literature phenotypes. Because the ensemble is being screened using the effect of different enzyme overexpressions on the same reference state, the final screened ensemble is independent of the path chosen, and can be represented by the intersection of the subsets screened by each phenotype. For demonstration, the results of screening for the reference flux with a glycolysis∶pentose phosphate pathway split ratio of 75∶25 are shown. The values indicate how many of the original ensemble of *n* = 1500 models match the given phenotype.

While we are screening from the previously determined subset, each of the perturbations was done in parallel, not sequentially. The screening for the Tkt and Tal phenotypes could be done independently, and the models common to both screens could be retained. Further, the order in which the models are screened does not influence the final number of models that matches all phenotypes. When the phenotypes are observed relative to the same reference state (i.e. done in parallel), they can all be viewed independently, and the models retained after screening can be illustrated as the intersection between each phenotype, as demonstrated in **Figure 5**.

From the reduced subset matching the Tkt and Tal phenotypes, screening for Pps overexpression leading to no change in DAHP production results in 8, 0, 318 and 278 models matching the phenotype for each of the split ratios. Of these models, the number that exhibited the property that the glucose uptake rate did not change when Tkt was overexpressed reduced the size of the ensembles to 7, 0, 241 and 235 models, in order of increasing glycolytic flux.

The final literature observation used for screening is that of an increase in DAHP production via Tkt overexpression being greater than the increase observed from Tal overexpression. This led to a final ensemble size of 171 and 195 models for the 75∶25 and 95∶5 split ratios, respectively (**Figs. 4C & 4D**). For the split ratios with a higher percentage of the flux through the pentose-phosphate pathway (**Fig. 4A & 4B**), zero of the models in the original ensemble remained after all of the screening steps. In fact, no models remained after just three screening steps when the flux was split 50∶50 between glycolysis and the pentose pathway. This indicates that in the EM approach, just as in nature, the effect of enzyme overexpression is dependent on the reference flux distribution. Further, this demonstrates that in the true system, the reference steady state flux may indeed have the majority of the metabolic flux directed through glycolysis, which follows the experimental observation that the overexpression of Tkt is limiting, as the Tkt net flux only becomes negative (thus feeding, not draining, the E4P pool) when the split ratio is 60∶40 or greater.

### Prediction of Tkt/Pps Dual Overexpression

**Figure 6:**
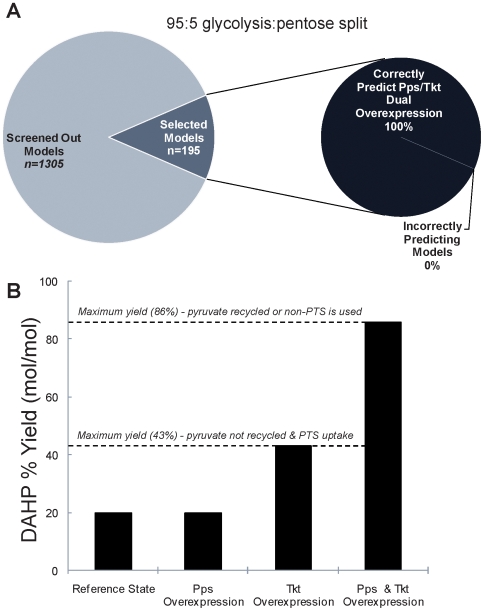
Dual overexpression kinetic phenomena for DAHP production. A) Illustration of the prediction for the dual overexpression of Tkt and Pps for the split ratio of 95∶5. 100% of the selected models exhibit the phenotype where when Tkt and Pps are simultaneously overexpressed, the combined effect is greater than the sum of the two individual overexpressions. B) The kinetic phenomenon in DAHP production illustrated. Pps overexpression does not increase DAHP production until Tkt is overexpressed. After removing this limitation, Pps overexpression has a dramatic effect on DAHP production, pushing yields near the theoretical limit of 86% mol DAHP/mol glucose.

To determine whether the screened ensemble of models becomes increasingly predictive, we test the behavior of the remaining models when both Tkt and Pps are simultaneously overexpressed two-fold. Indeed, even though these subsets of the original ensemble demonstrate that the sole overexpression of Pps has no effect on the DAHP steady state production rate, when Pps is overexpressed simultaneously with Tkt, 100% of the models for both the 75∶25 and 95∶5 split ratios yield an increase in the DAHP rate that is greater than the sum of the single overexpressions of Tkt and Pps, as shown in **Figure 6A**.

Since PEP is used in glucose transport via the PTS to form pyruvate, most carbon is not used in aromatics biosynthesis, resulting in a low yield. This problem was identified previously [22] and two solutions were developed. The first is recycling pyruvate back to PEP via overexpression of Pps, and the second is the use of non-PTS genes for glucose transport [25]. Interestingly, relieving this stoichiometric limitation did not increase the yield of DAHP until transketolase (the product of the *tktA* gene) was overexpressed [22], [26]. Apparently, a kinetic limitation caused by Tkt is the first bottleneck for DAHP production. Such behavior cannot be predicted using either stoichiometric models or a direct flux comparison between flux states. However, the EM approach was able to reproduce this phenomenon, which is illustrated in **Figure 6B**.

### Properties of Screened Models

**Figure 7:**
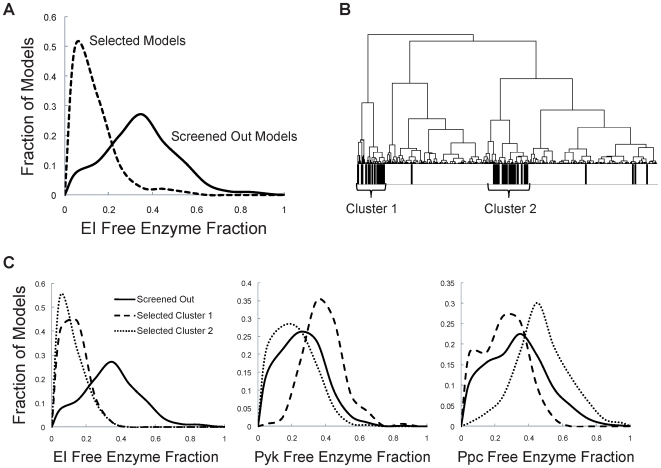
Analysis of parameters in screened models relative to original ensemble. Parameter analysis for the glycolysis∶pentose phosphate pathway split ratio of 95∶5. A) The distribution of the enzyme fraction representing the free enzyme EI for the original ensemble (solid line) compared to the final screened ensemble of *n* = 195 models (dashed line). B) Hierarchical clustering by the sampled enzyme fractions indicates that the screened models (denoted by black bars) exist primarily in two distinct clusters. C) The distribution of enzyme fractions representing the free enzymes EI, Pyk & Ppc for each of the two clusters (dashed lines) relative to the original ensemble (solid line). Both clusters have distributions in EI that deviate significantly from the original ensemble. Only cluster 1 has a distribution for Pyk significantly different from the original ensemble, and only cluster 2 has a Ppc distribution that deviates from the original ensemble.

For the final ensembles of 171 and 195 models for the split ratios of 75∶25 and 95∶5, respectively, the sampled parameters of these screened subsets were examined to determine what characteristics they exhibited that deviated from the original ensemble of models. The enzyme fractions of each free enzyme (enzyme not in a complex), and the net reversibilities of each overall reaction were examined to determine how their distributions for the screened models deviated from the original ensemble. For each parameter of interest, a two-sample Kolmogorov-Smirnov test [28] was conducted to determine if the distribution of that parameter within the screened models exhibited a significantly shifted distribution (with 95% confidence) when compared to the original ensemble of 1500 models. Examples of these distributions can be seen in **Figure 7**.

For both flux states examined, it was found that three of the enzymes showed a significant deviation in their distributions relative to the original ensemble, and none of the reaction reversibilities exhibited a deviation. For both flux states, the free enzyme fractions of enzyme I (EI), pyruvate kinase (Pyk), and phosphoenolpyruvate carboxylase (Ppc) had a significantly different parameter distribution compared to the original ensemble. **Figure 7A** demonstrates the change in the distribution of the EI free enzyme fraction for the 95∶5 split ratio. Each of these three identified enzymes share the metabolite phosphoenolpyruvate (PEP) as a reactant, which is also involved in the condensation reaction to form our desired product DAHP. Biologically, in the case of EI, the relatively low free enzyme fraction in the selected models indicates that glucose uptake is essentially saturated, and that a change in the concentrations of substrates of this reaction (such as an increase in the PEP concentration through overexpression of Pps) would have little effect on changing the system.

After determining that these three parameters were significantly distributed in the screened models, a hierarchical clustering analysis was performed examining these three free enzyme fractions in the original ensemble of models for the 95∶5 glycolysis∶pentose pathway split, as shown in **Figure 7B**. The models which were eventually to be selected from this ensemble existed in primarily two distinct clusters. To determine the differences between the models within these two clusters, the distribution of the parameter values in each individual cluster was calculated, and compared to the original ensemble (**Fig. 7C**). While both clusters show a similar distribution for the EI free enzyme fraction, the two clusters demonstrate significant differences when the Pyk and Ppc free enzyme fractions are compared. The Pyk free enzyme fraction of cluster 1 deviates from both the original ensemble and cluster 2, as cluster 1 has a higher proportion of the Pyk enzyme in its free form. The Ppc free enzyme fraction for cluster 2 is higher than both the original ensemble and cluster 1. Each of these enzymes reacts to form a complex with PEP, and thus by having a higher fraction in the free enzyme form, would leave more PEP free, keeping this metabolite from becoming limiting, and allowing these models to match the experimental phenotype indicating that E4P, and not PEP, is the first limiting metabolite in DAHP synthesis. Thus, there exist two alternative routes to match the experimentally observed phenotypes. Relatively high free enzyme fractions of Pyk and Ppc seem to act as alternative methods to achieve the observed phenotypes. If the free enzyme fractions of Pyk and Ppc are too low, much of the PEP concentration will be trapped in complex with the enzyme, and the PEP pool will become limiting. Further, if these two reactions have low free enzyme fractions, they are essentially saturated, and thus when Pps is overexpressed, there would be an increase in the PEP pool that would necessarily lead to an increase in DAHP production, as there would be no other alternative pathways to consume the increase in PEP.

### Spanning the Kinetic Space

**Figure 8:**
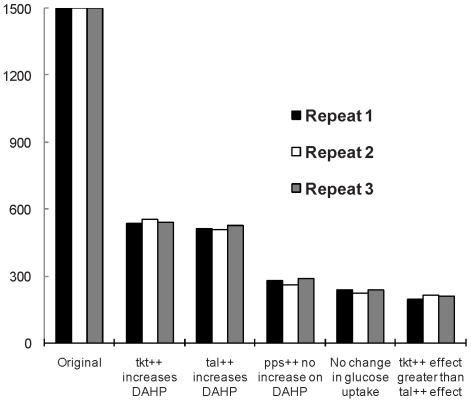
Repeatability of Ensemble Modeling. Three repeats of 1500 models in each ensemble for a 95∶5 split ratio show very similar models retained after each screening step. Less than 10% variance in the number of models retained is observed at each screening step, with each ensemble selecting out 195, 212, and 207 models, respectively, after all screening.

To determine if the construction of 1500 models in the original ensemble was enough to adequately cover the range of possible kinetics, and thus yield screening results which were reproducible, the ensemble construction and screening process for the 95∶5 split ratio was repeated three times to see the variability in the screening results. From an initial ensemble of 1500 models for each of the three ensembles, both the final number of models retained after all screening steps, and the models retained after each individual step varied less than 10% from repeat to repeat, shown in **Figure 8**. The final ensemble of models for the 95∶5 split ratio for each of the three repeats was 195, 207, and 212 models, indicating that the construction of 1500 models adequately covers the kinetic space.

## Discussion

In this work, we used the EM approach [11] to model the production of aromatic precursor DAHP production in *E. coli*. The difficulty in developing kinetic models for metabolic systems is well recognized, and is due to a lack of kinetic parameters. In this work, rather than attempting to construct a traditional kinetic model that matches dynamic metabolite concentration data and facing the issue of kinetic parameter identification, we focused on utilizing enzyme overexpression phenotype data, which are plentiful and relatively straightforward to acquire, to screen models. The EM approach is used to construct an ensemble of models for four different flux distributions, which are then screened using enzyme overexpression data from the literature. We show that in five screening steps, the ensembles can converge to a set of models which becomes predictive.

Since the entire flux map is not known, but only the external fluxes have been measured, a variety of split ratios were examined between glycolysis and the pentose phosphate pathway. The E4P metabolite pool is fed by transketolase (Tkt) “running backwards” from glycolysis, thus supporting the literature phenotypes only when the fraction of the carbon flux through glycolysis is above 60%. Interestingly, the EM approach was able to identify this property, as neither of the flux maps with less than a 60∶40 split ratio were able to retain any models that matched each of the screening phenotypes. However, many models were retained for each of the two flux distributions with greater than a 60∶40 split ratio. This indicates that a general idea of the flux distribution could be reverse engineered through the use of enzyme overexpression phenotypes and EM.

As one looks at the advantages and challenges of such an approach, it can be seen that for complicated networks with many pathways that lead to the external fluxes, there may be many degrees of freedom in determining the steady state flux, and thus the determination of the reference flux (the algorithm's primary input) may limit the possible systems to be studied. The iterative screening approach demonstrated here allows for the rapid collaboration and iteration between experiment and computation. As demonstrated for DAHP production, *any* enzyme overexpression phenotype can be used as a step in screening the ensemble, making *all* experimental data potentially useful, whether the data shows an increase in the production rate of interest, a decrease, or no change at all. While the predictive capabilities of the EM approach are primarily qualitative, this provides a tool to drive experimentation and to learn from the results. If a prediction is proven to be incorrect, this information can also be incorporated into the screening, allowing for the formation of an alternate hypothesis.

In using reported enzyme overexpression data for Tkt, Tal and Pps, it is demonstrated that the screened set of models exhibits the kinetic phenomena that is at work in the network, without the need for kinetic parameters. The final screened ensemble of models shows that Tkt is the first limiting step, and correctly predicts that only after this limitation is lifted does an increase in Pps increase the production rate of DAHP. This work thus demonstrates that EM is able to capture kinetic properties of metabolic networks by utilizing enzyme tuning data to refine and screen the ensemble.

## Methods

### Obtaining Steady State Fluxes

The first step in EM is to obtain the steady-state fluxes in the reference state of interest. The reference state is typically the base-line control strain before further metabolic engineering. In order to model a given steady state, the flux distribution of that state must first be determined. This flux distribution, or flux map, can be deduced via a variety of methods. Typically, the external fluxes of the system may be known, or easily measureable using GC or HPLC. In this case, the internal fluxes of the system can be estimated by a standard flux balance around each metabolite at the steady state:
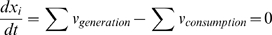
(1)


This can be represented for the entire network in matrix form:
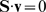
(2)where the matrix **S** is the *m* x *n* stoichiometric matrix consisting of *m* metabolites and *n* net reactions, and **v** is the *n* x 1 vector of net reaction rates. For more detailed analysis, the full flux map may be determined through the use of C13 isotopomer analysis [29]–[33], which involves feeding the cells a precisely labeled mix of glucose. However, such detailed analyses are not necessary in the first step.

### Model Building Using Elementary Reactions

At the molecular level, the basic elementary reactions (either bi-molecular or uni-molecular) that follow mass-action kinetics are the most fundamental kinetic events [6]. An elementary reaction is a chemical reaction in which one or more of the chemical species react directly to form products in a single reaction step and with a single transition state. These reactions follow mass action kinetics. Free enzymes and enzyme-substrate complexes are treated as separate chemical species as either reactants or products. The elementary reactions are the basis for deriving lumped enzyme kinetic rate laws such as Michaelis-Menten kinetics and more complicated allosteric regulation kinetics. Because of total enzyme conservation, the elementary reactions automatically give rise to a saturation behavior characteristic in biological reactions. Regulatory mechanisms are also broken down in terms of elementary reactions. In the EM approach, each enzymatic reaction is broken down into a series of elementary reactions, thus preserving the fundamental behavior we know to exist in metabolic networks. The mechanism that is used for the various metabolic reactions in this work is depicted in **Figure 1**, for various combinations of reactants and products. The PTS is modeled as a series of phosphorylation steps [11]. For modeling purposes, each of the phosphorylated enzymes is considered to be a substrate or reaction product, while each of the free enzymes is considered to be the enzyme that participates in the reaction. Thus, for the entire PTS, each enzyme drives a step of the reaction and is then regenerated in the subsequent phosphorylation step. Also demonstrated in **Figure 1** is how regulatory steps can be easily included into the framework.

The general form for the elementary reactions in an enzymatic reaction of one substrate to one product can be depicted as follows:
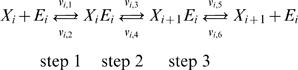
where the rate of each individual elementary reaction, 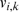, follows the mass action principle:
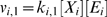
(3)where 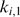 is the rate constant of the forward reaction of step 1 of the overall reaction catalyzed by the enzyme *i,* 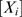 is the concentration of metabolite *i*, and 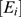 is the concentration of free enzyme *i*. To avoid quantifying the absolute concentrations of each metabolite and enzyme, we scale their concentrations by the corresponding concentration at the steady state, and Eq. (3) becomes:
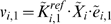
(4)where 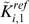 is the rescaled kinetic parameter defined as:
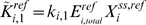
(5)with
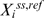
equal to the metabolite's concentration at steady state, and 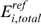 the total concentration of the corresponding enzyme at the reference state. Note that the rate law in Eq. (4) has the log-linear form:
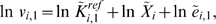
(6)


Each of the transport reactions out of the system are modeled with mass-action kinetics:
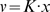
(7)


For the reference state, 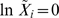, and 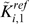
can be calculated from Eq. (6) after the uni-directional flux 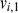 and enzyme fraction 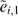 are determined. The uni-directional flux 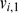 is determined from the sampled reversibility (see next section), and the enzyme fraction 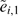 is sampled directly (see next section).

### The Ensemble Modeling (EM) Sampling Algorithm

The EM methodology is illustrated in **Figure 2**. The inputs into the algorithm are the steady state flux distribution of the reference state and the Gibbs free energies of the reactions, which allow for the assignment of thermodynamic constraints on the system. The reference steady state fluxes are obtained as described above, while the range of Gibbs free energies are calculated from the standard Gibbs free energies [34] allowing for a one-hundred fold change in metabolite concentrations.

In the first sampling step, the reversibilities for each elementary step 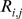 are sampled. These reversibilities can be related to the individual elementary reaction rates via the following:
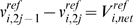
(8)

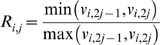
(9)where 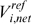 is the net flux of the reaction *i* at the reference steady state, and 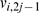 and 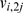 are the forward and backward rates of step *j* in reaction *i*. The reversibilities range from 0 (for an irreversible step) to 1 (for a step at equilibrium). Thus, the forward and backward rates can be calculated from the reversibility and the steady state flux determined in the first step:



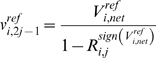
(10)

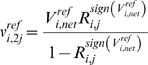
(11)where *sign(V_i,net_)* represents the direction of the net flux (positive if forward and negative if backwards). The reversibilities are constrained by the Gibbs free energy of the overall reaction, 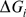:
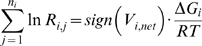
(12)where *n_i_* represents the number of elementary steps for enzyme *i*. This requires that the net flux of reaction *i* be positive if 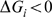, and negative if 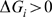. This constraint is used to check if the reference steady-state is thermodynamically compliant. Since the exact values for the Gibb free energies are not known, but their ranges can be determined [34]–[36], Eq. (12) at the reference state becomes:
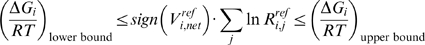
(13)


This relationship between the reversibilities of the elementary steps, the net reaction, and the free energy bounds has been previously derived [11].

Next, the enzyme fractions are sampled. The enzyme fractions are the fractions of the total enzyme amount that exist as the free enzyme and as the various enzyme complexes at steady state, such that the sum of all the fractions are equal to one, conserving the total amount of each enzyme:
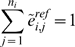
(14)


At the reference steady state 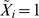 and Eq. (6) becomes:
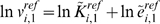
(15)


Once this sampling of enzyme fractions is complete, the kinetic parameters for that model can be assigned. The kinetic parameters 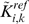 can be easily computed from Eq. (15), as 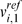
is determined from the sampling of reversibilities (Eqs. (10) and (11)), and 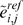 is directly sampled.

### Establish the Ensemble of Models

Next the ordinary differential equations (ODEs) governing the system are solved at the reference state. The network can be described by a system of ODEs and solved numerically, where the metabolite concentrations and enzyme fractions, not the total enzyme concentrations, are the ODE variables:
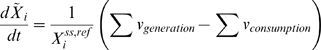
(16)

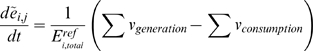
(17)


With enzyme fraction initial conditions set such that:
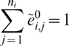
(18)where the superscript “0” represents the initial condition of the enzyme fractions.

Different combinations of reversibilities represent different kinetic states. Each model is a function of the reversibilities and enzyme fractions:
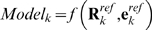
(19)


Every model reaches the same steady state, and the reversibilities 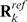 (which represents the vector composed of the reversibilities for each elementary step) and enzyme fractions 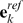
(which represents the vector composed of the enzyme fractions for each free enzyme and enzyme complex) are reassigned for each subsequent model. This allows for the formation of an ensemble of models that span the range of kinetics allowable by thermodynamics.

### Screening of the Models by Perturbation

The entire ensemble can be perturbed to determine each individual model's response to the enzyme expression perturbation with which we would like to screen the ensemble. In order to perturb the concentration of an enzyme for an individual model in the ensemble, Eq. (4) is modified:
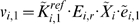
(20)


The additional variable 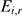 represents the fold change in total enzyme concentration relative to the reference state. Each enzyme of interest is overexpressed *n*-fold (*E_i,r_ = n*) to determine its effect on production. Here we use *n* = 2. If the metabolic network contains any moiety conservation relationships [37], the initial conditions are set such that the sum of these metabolite remain unchanged. For example, the sum of cofactors and their intermediates in the new perturbed condition must be equal to those in reference steady state.
